# Effect of sildenafil on platelet activation and mediators of vascular remodelling during LVAD support

**DOI:** 10.1093/eschf/xvag099

**Published:** 2026-04-02

**Authors:** Omar Saeed, Snehal R Patel, Muhammad Farooq, Morayma Reyes Gil, Clemencia Solorzano, Freda Afrifa, Xiaonan Xue, Julio Ovalle Ramos, Sasa Vukelic, Shivank Madan, Yogita Rochlani, Sandhya Murthy, Daniel B Sims, Julia Shin, Daniel J Goldstein, Nicolas Sibinga, Karina Yazdanbakhsh, Jorge R Kizer, Ulrich P Jorde

**Affiliations:** Department of Medicine, Division of Cardiology, Montefiore Medical Center, Albert Einstein College of Medicine, 3400 Bainbridge Avenue, NewYork, NY 10467, USA; Department of Medicine, Division of Cardiology, Northwell Health, Long Island, NY, USA; Department of Medicine, Division of Cardiology, Montefiore Medical Center, Albert Einstein College of Medicine, 3400 Bainbridge Avenue, NewYork, NY 10467, USA; Department of Pathology, Cleveland Clinic Foundation, Cleveland, OH, USA; Department of Pharmacy, Montefiore Medical Center, Albert Einstein College of Medicine, NewYork, NY, USA; Department of Pharmacy, Montefiore Medical Center, Albert Einstein College of Medicine, NewYork, NY, USA; Department of Epidemiology and Population Health, Albert Einstein College of Medicine, NewYork, NY, USA; Department of Medicine, Division of Cardiology, Montefiore Medical Center, Albert Einstein College of Medicine, 3400 Bainbridge Avenue, NewYork, NY 10467, USA; Department of Medicine, Division of Cardiology, Tulane University, New Orleans, LA, USA; Department of Medicine, Division of Cardiology, Montefiore Medical Center, Albert Einstein College of Medicine, 3400 Bainbridge Avenue, NewYork, NY 10467, USA; Department of Medicine, Division of Cardiology, Montefiore Medical Center, Albert Einstein College of Medicine, 3400 Bainbridge Avenue, NewYork, NY 10467, USA; Department of Medicine, Division of Cardiology, Montefiore Medical Center, Albert Einstein College of Medicine, 3400 Bainbridge Avenue, NewYork, NY 10467, USA; Department of Medicine, Division of Cardiology, Montefiore Medical Center, Albert Einstein College of Medicine, 3400 Bainbridge Avenue, NewYork, NY 10467, USA; Department of Medicine, Division of Cardiology, Montefiore Medical Center, Albert Einstein College of Medicine, 3400 Bainbridge Avenue, NewYork, NY 10467, USA; Department of Cardiothoracic and Vascular Surgery, Montefiore Medical Center, Albert Einstein College of Medicine, NewYork, NY, USA; Department of Medicine, Division of Cardiology, Montefiore Medical Center, Albert Einstein College of Medicine, 3400 Bainbridge Avenue, NewYork, NY 10467, USA; NewYork Blood Center, New York, NY, USA; Cardiology Section, San Francisco Veterans Affairs Health Care System, and Departments of Medicine, Epidemiology, and Biostatistics, University of California San Francisco, SanFrancisco, CA, USA; Department of Medicine, Division of Cardiology, Montefiore Medical Center, Albert Einstein College of Medicine, 3400 Bainbridge Avenue, NewYork, NY 10467, USA

**Keywords:** Platelets, LVAD, Sildenafil

## Abstract

**Introduction:**

Observational studies provide a signal that phosphodiesterase-5 inhibitors such as sildenafil are associated with lower mortality and ischaemic stroke during durable left ventricular assist device (LVAD) support. This study aims to determine the causal effects of sildenafil on platelet activation and circulating mediators of vascular remodelling during LVAD support.

**Methods:**

We conducted a double-blind, randomized, placebo-controlled study to determine the effect of sildenafil on platelet activation and circulating mediators of vascular remodelling. Stable participants on LVAD support were assigned to sildenafil or placebo every 8 h for a 15-day period. Three primary endpoints were percent change in platelet activation by collagen, thromboxane (Tx) A2, and adenosine diphosphate (ADP). Secondary endpoints included changes in circulating mediators of vascular remodelling.

**Results:**

Twenty participants were randomized. On day 15, those on sildenafil had lower collagen (−41%, −60 to −24, vs. −7%, −18 to 79, *P* = .038), but not TxA2 (−24%, −45 to −14 vs. 3%, −15 to 87, *P* = .112), and ADP (−5%, −70 to 25 vs. 31%, −29 to 242, *P* = .122) induced platelet activation compared to placebo. Endothelin-1 changed by −30% (−53 to 4) in the sildenafil group vs. −3% (−18 to 22) with placebo (*P* = .041). Angiopoietin (ang)-2 changed by −5% (−11 to −4) with sildenafil compared to 3% (−5 to 15) with placebo (*P* = .039), while ang-1 increased by 24% (17–54) with sildenafil and was −4% (−19 to −2) with placebo (*P* = .001), leading to a change in the ang-2/ang-1 ratio by −23% (−45 to −15) with sildenafil compared to 8% (−3 to 24) with placebo (*P* = .001). Hs-CRP and fibrinogen were unchanged between the groups.

**Conclusion:**

Fifteen days of sildenafil exposure modifies platelet activation and lowers mediators of vascular remodelling on LVAD support.

## Graphical Abstract

**Graphical Abstract:**
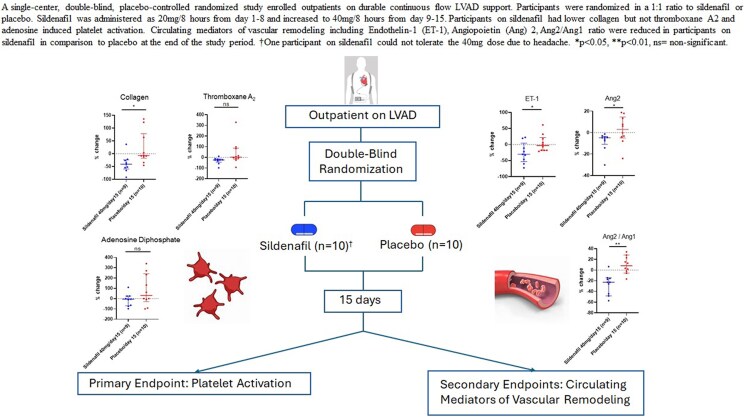

## Introduction

Left ventricular assist device (LVAD) therapy is a key life-extending option for patients with advanced heart failure (HF),^1^ thereby making it imperative to develop strategies to improve its durability and adverse event profile. Although contemporary LVAD therapy improves survival, serious adverse events originating from macro- and microvascular systems, such as stroke and bleeding, occur in >40% of the patients over 5 years.^2^ Stroke is a major cause of death after LVAD placement, as survival after stroke drops by nearly 50%.^3^ Moreover, stroke of any severity markedly increases disability, limits functional capacity, and drastically reduces the quality of life for patients with LVADs and may impact the eligibility for heart transplantation in the future.^3^ Meanwhile, nonsurgical bleeding, which commonly originates from gastrointestinal angiodysplasia, is the culprit in 22% of rehospitalizations in the 2 years following LVAD placement.^4^

Platelet activation and vascular remodelling are exacerbated in patients on contemporary LVAD support, and these pathways may contribute to an elevated burden of stroke and bleeding. Histological analysis of paired aortic samples before and after LVAD placement shows rapid arterial wall thickening with collagen deposition and fibrosis.^5^ At microvascular levels, dysplastic angioectasia formation is mediated by elevated angiopoietin (ang) 2, which triggers abnormal vascular growth in association with inflammation on LVAD support.^6^ Phosphodiesterase-5 inhibitors (PDE5i), such as sildenafil, enhance nitric oxide (NO) signalling and are clinically used in select patients on LVAD support with right heart dysfunction and pulmonary hypertension. Due to additional antithrombotic,^7–9^ antifibrotic,^10–13^ and anti-inflammatory^10^ properties, PDE5i could potentially limit vascular remodelling and related adverse events. Accordingly, in a single-centre observational study, we noted that sildenafil use was associated with a lower risk of pump thrombosis and ischaemic stroke during Heart Mate (HM) II support in a subgroup of at-risk patients with low levels of haemolysis.^14^ Subsequent Interagency Registry for Mechanically Assisted Circulatory Support (INTERMACS) analyses showed that this association was present across a spectrum of LVAD types, including HM II, HeartWare (HVAD), and HM 3.^15,16^ Moreover, these studies from a large and contemporary cohort of patients also noted lower mortality with sildenafil use during HM 3 support.^16^

Despite favourable clinical associations, it is not known whether sildenafil can limit platelet activation and mechanisms of vascular remodelling during LVAD support. To determine the effect of sildenafil on these pathways, we performed the following investigation in stable and late survivors with ongoing LVAD support.

## Methods

A single-centre, double-blind, placebo-controlled study was conducted to determine the effect of sildenafil administration on platelet activation and mediators of vascular remodelling during LVAD support. The eligibility criteria were as follows: (i) stable outpatients on continuous flow (CF) LVAD support over 18 years of age, (ii) not clinically receiving sildenafil or any other PDE-5i, (iii) not clinically on nitric oxide donor medications, and (iv) no active infection. With the utilization of HM 3, which produces lower levels of lactate dehydrogenase (LDH) than HM 2, the inclusion criteria for LDH thresholds that were originally considered at study conception were not applicable and were removed prior to study initiation. Study participants were enrolled after giving informed consent. Baseline characteristics and blood samples were collected. Then, participants were randomized in a 1:1 ratio to either sildenafil or a matching placebo and received their assigned study drug in a blinded manner. Study drug allocation was concealed from all members of the study team. The randomization list was held by the investigational pharmacist dispensing the study drug and who was not involved in any study-related procedures or participant interaction. Randomization was performed in blocks of two participants and was not stratified by device type. Enrolment into the study was conducted from 3 June 2019 to 20 January 2023. The study was registered on clinicaltrials.gov (NCT03199612) and approved by the institutional review board of Montefiore Medical Center at the Albert Einstein College of Medicine (2016–7404).

### Study drug administration

After collecting baseline blood samples, we administered 20 mg of the study drug on day 1, and blood pressure was monitored by brachial artery Doppler ultrasound for 2 h. If this opening Doppler blood pressure (DopBP) remained stable without a >5 mmHg drop, then participants proceeded to take 20 mg of the study drug every 8 h (study visit 1). Participants returned to the study site on day 8 and received a 20 mg dose of the study drug. Blood samples were collected after 2 h, and participants resumed the study drug at 20 mg every 8 h for the next two doses (study visit 2). The participants returned to the study site for study visit 3 on day 9 and received 40 mg of the study drug. If blood pressure remained stable without a >5 mmHg drop, then participants proceeded to take 40 mg of the study drug every 8 h. The study participants returned to the study site on day 15 for the final study visit 4. They received 40 mg of the study drug, and blood samples were collected after 2 h. Close monitoring for adverse events was performed during the 15-day enrolment period. Pill counts were performed to confirm adherence to the study drug. An independent and external data and safety monitoring board (DSMB) reviewed quarterly reports throughout the study period. We assessed the effects of 20 and 40 mg doses, since sildenafil is commonly prescribed in this range to patients supported by durable LVADs with right heart dysfunction and pulmonary hypertension.

### Study measures and power

The study included three primary endpoints, which were the percent change in platelet activation by collagen, thromboxane (Tx) A2, and adenosine diphosphate (ADP) at day 15 between groups. To determine the effect of sildenafil or placebo on platelet activation and aggregation, we utilized *ex vivo* whole blood aggregometry (Chronolog Model 700) as described previously.^17^ Whole blood samples were exposed to the following platelet agonists: (i) collagen (5 µg/ml), (ii) TxA2 (10 µg/ml), and (iii) ADP (10 µM). A stable analog for TxA2 (U-46619, Sigma) was utilized, and the dose was informed by prior clinical experience. After exposure to a platelet agonist, platelet activation and aggregation were quantitated by the area under the curve (AUC). AUC was calculated as resistance (ohms) × time (s). Platelet activation and aggregation was assessed for 14 min. Higher AUC indicates greater platelet activation and aggregation. Study samples were processed within 60 min from blood draw and remained in room temperature. The aggregometer was calibrated prior to sample processing with verification of baseline impedance, temperature, and zero-point. Quality control was performed by the coagulation laboratory staff on a routine basis using manufacturer-recommended reagents as the aggregometer was also utilized for clinical testing. With performance of 4-channel aggregometry and serial testing, ample sample volume for replicate testing was not obtainable from this vulnerable study population. All samples had platelet counts within a reliable device operational range of >150 000/µl and <450 000/µl. Laboratory staff were blinded to treatment allocation. As an internal control, we also measured platelet activation after exposure to arachidonic acid (AA), which specifically assesses aspirin-related platelet inhibition. Aspirin blocks cyclooxygenase (COX)-1, which is needed for AA to activate platelets; hence, aspirin users would not be expected to show platelet activation by AA. Moreover, AA would only activate platelets in participants not taking aspirin. Since the participant’s aspirin use status was known, if platelet aggregometry was functioning correctly, then only those participants not on aspirin would have platelet activation by AA. Aspirin users took their usual aspirin dose within 24 h prior to blood sampling. Blood samples were obtained at 8–10 am in a fasting state, and study participants were advised to refrain from smoking tobacco throughout the enrolment period. Based on prior studies in non-LVAD populations, the study was designed to enrol 46 total patients to have 85% power for detecting a 40% reduction in platelet activation with sildenafil.

Enrolment into the study was slow due to constraints imposed by (i) the COVID-19 pandemic and (ii) competing studies of antiplatelet therapy at our centre. Given these limitations in the pace of enrolment, only 20 participants were enrolled and completed the study within the funded study period. An interim analysis was performed to assess the percent change in platelet activation for the primary endpoints between groups. We then proceeded to perform a conditional power analysis with data from these 20 enrolled participants to determine the probability that study findings would remain similar if the original proposed sample size was enrolled. The study was then terminated.

As secondary endpoints, we determined the effect of sildenafil on mediators of vascular remodelling. We assessed circulating levels of endothelin (ET)-1 (lower detection limit: 0.2 pg/ml) and angiopoietin (ang)-1 (lower detection limit: 0.01 ng/ml), ang-2 (lower detection limit: 0.02 ng/ml), by enzyme-linked immunosorbent (ELISA) kits from Quantikine (Minneapolis, MN, USA). Assays were run in triplicate in a single batch. Intra-assay coefficients of variation were 3% (ET-1), 3% (ang-1), and 7% (ang-2), showing acceptable precision, and all samples were within detection limits. High sensitivity C-Reactive protein (hs-CRP) was measured by the Alinity Vario immunoassay (Abbott, Il, USA) and fibrinogen by the STA kit (Stago, Paris, France) as described previously.^18^

### Statistical analyses

For descriptive purposes, categorical variables are expressed as frequencies and percentages, which were compared with χ2 testing. Continuous variables are shown as median with Q1–Q3. Continuous variables were assessed for normality by the visual inspection of their distribution and q-q plots. Percent change in endpoints was compared between groups by the Mann-Whitney test for non-normal distributions and by the two-sample *t*-test for normally distributed variables. Multivariable linear regression was performed for endpoint measures with adjustment for covariates that had a *P* ≤ .10 between study groups, which was body mass index only. For multivariable linear regression, normality was assessed by visual inspection of variable distribution, along with the Shapiro-Wilk test, and non-normal distributions were log transformed. To perform log transformation, a constant of 110 was added to allow a shift to positive numbers. While platelet activation was originally conceived to be measured by ADP only, we added collagen and TxA2 agonists prior to study initiation, to improve the capability for detecting more changes in platelet activation by sildenafil. To adjust for multiple comparisons across endpoints of platelet activation, we corrected *P*-values for each agonist by the Benjamini-Hochberg (B-H) method with a false discovery rate of 0.05. Within-group changes in study endpoints at day 8 (20 mg dose) and day 15 (40 mg dose) from baseline were assessed by the Wilcoxon signed rank test. *P*-values of < .05 were considered statistically significant. Within-group supplemental analyses, restricted to only those participants on HeartMate (HM) 3 support assigned to sildenafil, were also performed. All statistical analyses were performed in Stata version 16.0. Figures were created in GraphPad Prism. Q1–Q3 for study measures were calculated in GraphPad Prism.^19^

## Results

### Baseline characteristics

**Figure 1:**
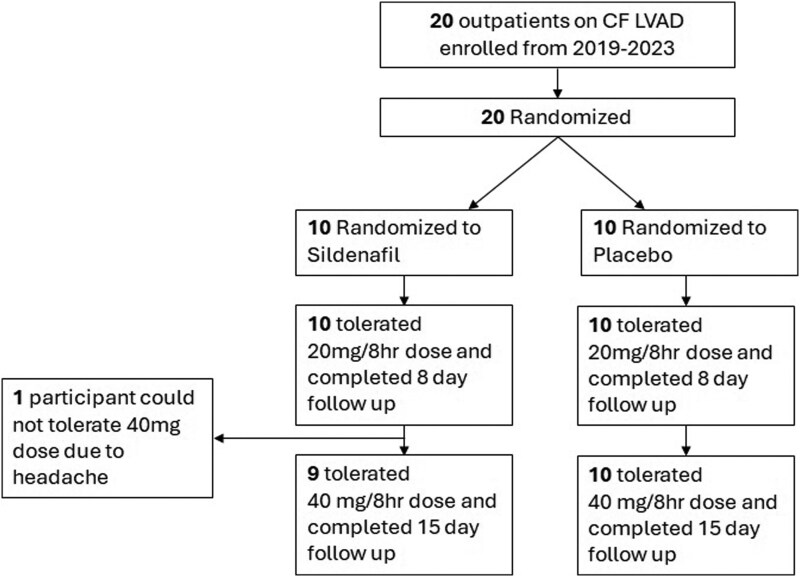
Consort diagram. CF LVAD, continuous flow left ventricular assist device

Overall, 20 participants were enrolled in the study (*Figure 1*). They were 55 (45–65) years old, and 4 (20%) were female. They had been on LVAD support for 658 (318–1430) days. Fifteen (75%) participants were on HM 3 support, while 5 (25%) had an HM II in place. Fourteen (70%) of the participants were clinically receiving aspirin 81 mg, and none of them had any changes in aspirin therapy during the enrolment period. INR was 2.2 (1.5–2.6) with 18 (90%) participants receiving anticoagulation with warfarin (*Table 1*).

**Table 1:** Baseline characteristics of study participants.

	All (*n* = 20)	Sildenafil (*n* = 10)	Placebo (*n* = 10)	*P* value*
**Age (years)**	55 (45–65)	55 (45–63)	56 (44–68)	.98
**Female (*n*, %)**	4 (20)	2 (20)	2 (20)	1.00
**Body mass** i**ndex (Kg/m^2^)**	26 (24–33)	30 (25–41)	25 (21–26)	.07
**Ischaemic cardiomyopathy (*n*, %)**	6	3	3	1.00
**Diabetes mellitus (*n*, %)**	8	4	4	1.00
**History of stroke (*n*, %)**	3	2	1	.39
**Race/ethnicity (*n*, %)**	NHB: 9 (45) NHW: 4 (20) H: 7 (35)	NHB 5 (50) NHW 1 (10) H: 4 (40)	NHB: 4 (40) NHW: 3 (30) H: 3 (30)	.53
**LVAD type**	15 HM3 5 HMII	9 HM3 1 HMII	6 HM3 4 HMII	.12
**LVAD duration (days)**	658 (318–1430)	395 (201–1197)	1117 (532–1614)	.11
**History of HRAE during LVAD (*n*, %)**	5 (25)	2 (20)	3 (30)	.61
**Aspirin (*n*, %)**	14 (70)	8 (80)	6 (60)	.31
**INR (IU)**	2.2 (1.5–2.6)	2.2 (1.9–2.5)	2.2 (1.1–2.7)	.74
**eGFR (ml/min/BSA)**	67 (52–87)	67 (53–89)	67 (51–85)	.85
**Lactate Dehydrogenase (U/l)**	262 (234–362)	244 (233–357)	301 (240–367)	.87
**H**a**emoglobin (g/dl)**	12.9 (11.5–14.0)	12.9 (12.1–13.6)	12.4 (10.5–14.1)	.47
**Platelets (k/ul)**	189 (169–260)	230 (169–293)	185 (168–189)	.23
**White blood cell (k/ul)**	6.1 (5.1–8.7)	7.3 (5.5–9.3)	5.8 (4.5–7.0)	.11
**Haptoglobin (mg/dl)**	61 (16–118)	101 (16–138)	41 (16–97)	.24

LVAD, left ventricular assist device; NHB, non-Hispanic black; NHW, non-Hispanic white; H, Hispanic; HRAE, hemocompatibility-related adverse events; INR, international normalized ratio; GFR, glomerular filtration rate; BSA, body surface area.

^*^ *P* value for sildenafil vs placebo.

Given the randomization scheme, 10 (50%) participants received sildenafil, and 10 (50%) were assigned to placebo. Baseline characteristics, including age (sildenafil: 55 (45–63) vs. placebo: 56 (44–68) years, *P* = .98), and the proportion of females [sildenafil: 2 (20%) vs. placebo: 2 (20%)] were similar between groups (*Table 1*). There were no differences in the change in INR or warfarin dose between groups during the enrolment period (Supplementary Figure S1A and B). LVAD speed was not changed for study participants during the enrolment period.

### Adverse events and blood pressure

No patients experienced any serious adverse events or hemocompatibility-related adverse events during the enrolment period. As noted in *Figure 1*, one participant could not tolerate 40 mg of sildenafil due to a headache. The remainder of the participants completed the 15-day enrolment period without any adverse events.

During the enrolment period, there were minimal changes in background heart failure and antihypertension medical therapies, with only one participant undergoing an increase in hydralazine dosage (Supplementary Table S1). On day 15, the change in DopBP from baseline was similar between patients on sildenafil (−2, −13 to 2 mmHg) vs. placebo (0, −2 to 5 mmHg, *P* = .22, Supplementary Figure S2A). Within groups, DopBP changed from 82 (80–91 mmHg) at baseline to 83 (80–96 mmHg, *P* = .28) on 20 mg and to 82 (80–87 mmHg, *P* = .44) on 40 mg sildenafil (Supplementary Figure S2B). No significant changes were noted in DopBP with placebo (Supplementary Figure S2B).

### Platelet activation and aggregation

**Figure 2:**
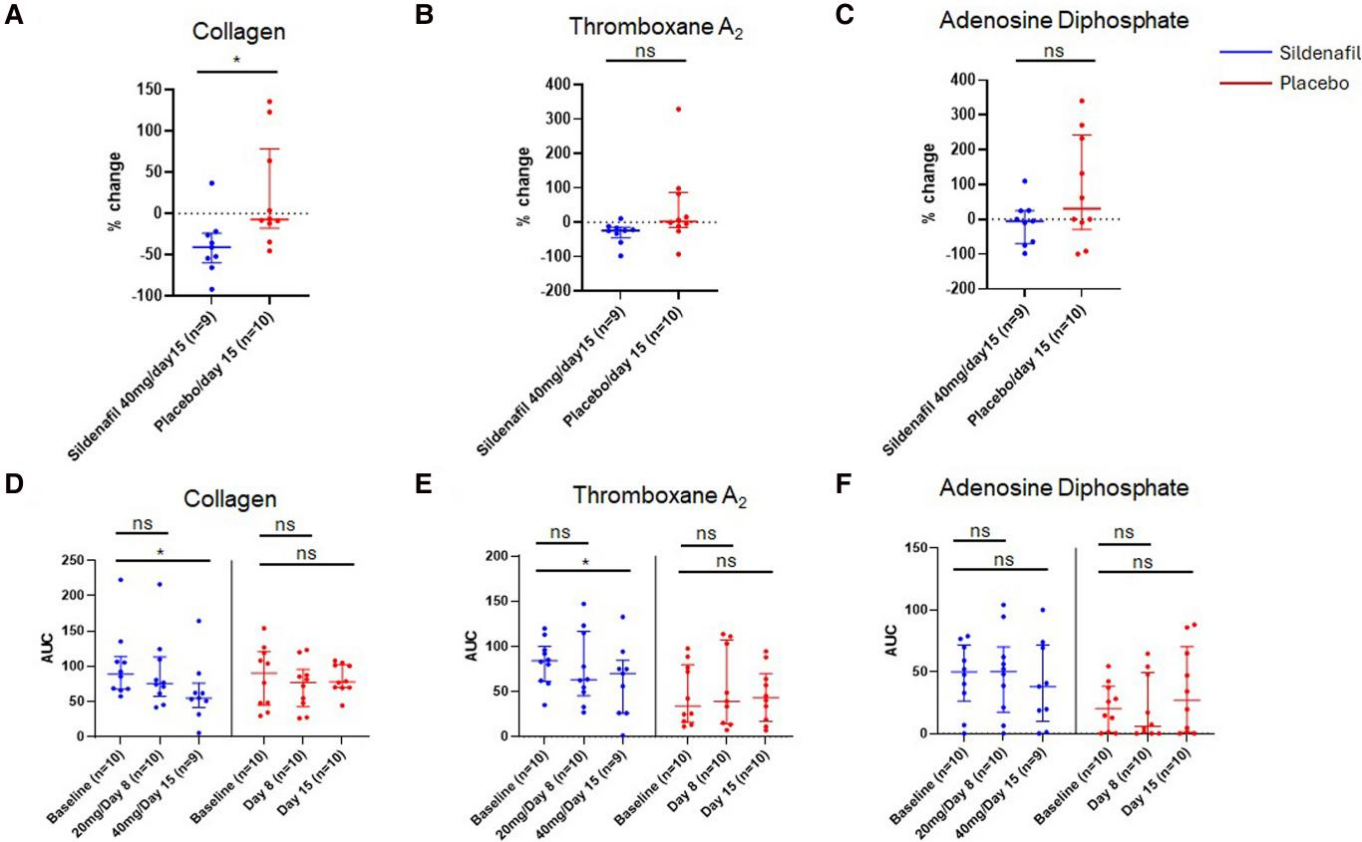
Between-group changes in platelet activation and aggregation from baseline to the end of the enrolment period on day 15 in participants on sildenafil and placebo. Platelet activation and aggregation was measured by *ex vivo* whole blood aggregometry with agonism from *A*) collagen, *B*) thromboxane A_2_, and *C*) adenosine diphosphate (ADP) and is shown as area under the curve (AUC). Between-group comparisons were adjusted for body mass index*. D-F*) Within group changes in platelet activation and aggregation across study time points. Data are shown as median (Q1–Q3), *P < .05, ns = non-significant.

Between groups, on day 15, participants on sildenafil had lower collagen (−41%, −60 to −24, vs. −7%, −18 to 79, B-H corrected *P* = .027, *Figure 2A*) and TxA_2_ (−24%, −45 to −14 vs. 3%, −15 to 87, B-H corrected *P* = .042, *Figure 2B*), but not ADP (−5%, −70 to 25 vs. 31%, −29 to 242, B-H corrected *P* = .220) induced platelet activation in comparison to placebo (*Figure 2C*). After adjustment for body mass index, there remained a significant difference only in percent change of collagen (B-H corrected *P* = .038), but not TxA_2_ (B-H corrected *P* = .112) and ADP (B-H corrected *P* = .112) induced platelet activation between participants on sildenafil and placebo.

Within groups, collagen-induced platelet activation and aggregation changed from 89 (68–114 AUC) at baseline to 76 (58–114 AUC, *P* = .08 vs. baseline) with 20 mg sildenafil and were reduced to 55 (42–76 AUC, *P* = .01 vs. baseline) with 40 mg sildenafil (*Figure 2D*). TxA_2_-induced platelet activation and aggregation were 84 (61–100 AUC) at baseline, 63 (45–117 AUC, *P* = .38 vs. baseline) with 20 mg sildenafil, and 70 (26–85 AUC, *P* = .02 vs. baseline, *Figure 2E*) with 40 mg sildenafil. In contrast, platelet activation and aggregation remained similar with ADP from 50 (33–72 AUC) to 50 (17–70 AUC, *P* = .81 vs. baseline) with 20 mg sildenafil and were 38 (10–72 AUC, *P* = .53 vs. baseline) with 40 mg sildenafil (*Figure 2F*). There were no significant changes in platelet activation and aggregation in patients on placebo during the study (*Figures 2D–F*). To confirm internal validity, participants receiving aspirin demonstrated no platelet activation after the addition AA (baseline: 0, 0–0 vs. 14, 5–33 AUC, day 8: 0, 0–0 vs.18, 5–59 AUC, day 15: 0, 0–0 vs. 16, 5–29 AUC), in contrast to those not on aspirin across study time points (Supplementary Figure S3).

### Conditional power

Based on the observed differences in platelet activation by collagen between the treatment groups, if the study had continued to its full enrolment of 23 patients in each arm and under the same alternative hypothesis of at least 40% reduction in platelet activation, the study had 95% probability of rejecting the null hypothesis of no treatment effect. The large conditional power suggests that findings showing a significant reduction in collagen-induced platelet activation after sildenafil administration are expected to remain similar if the study were finished as planned. There was no significant difference in platelet activation observed for TxA2 and ADP agonists between groups; hence, the study concluded no treatment effect for these endpoints.

### Mediators of vascular remodelling

**Figure 3:**
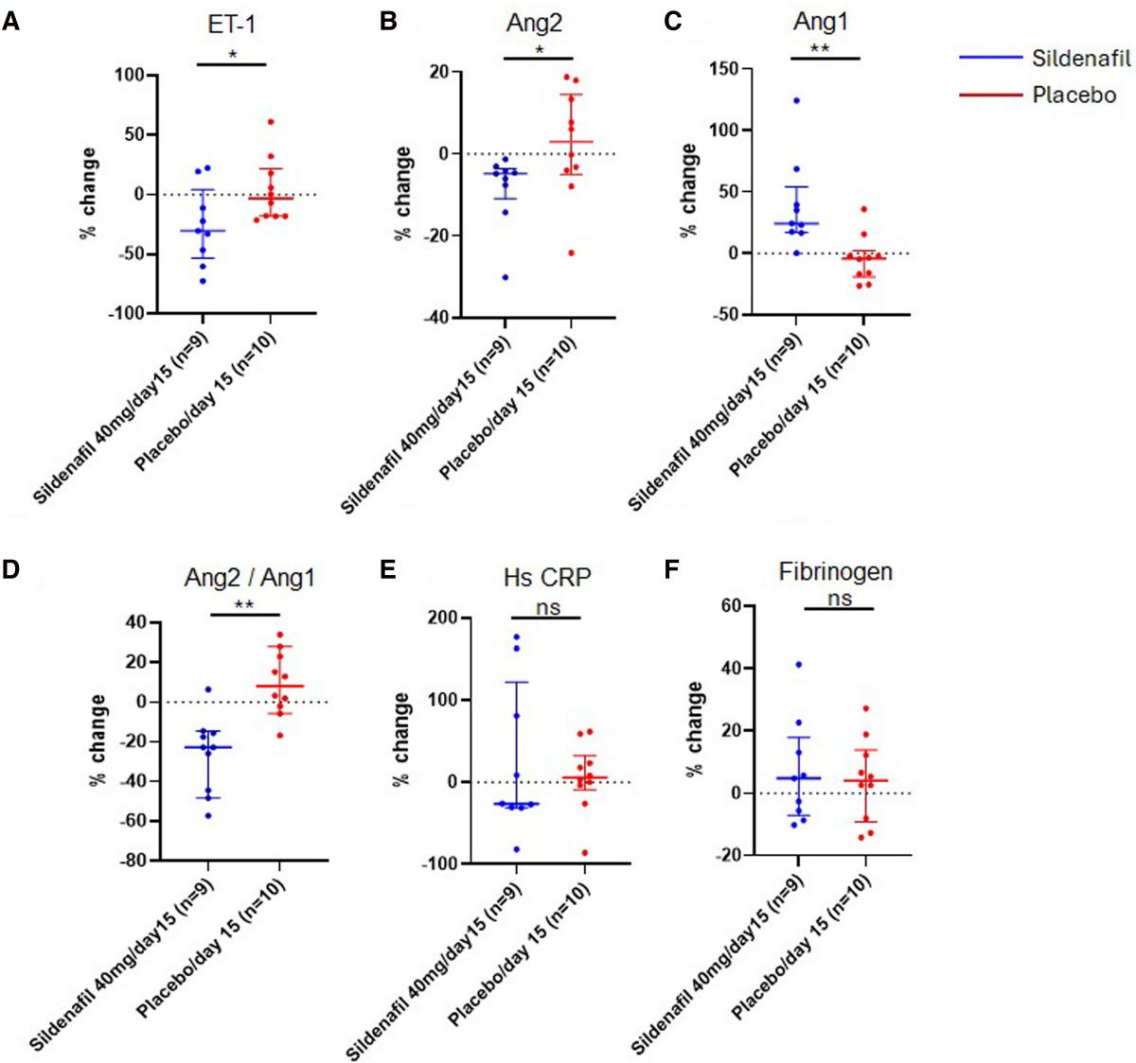
Between-group changes in circulating levels of *A*) endothelin-1 (ET-1), *B*) angiopoietin (Ang) 2, *C*) angiopoietin (Ang) 1, *D*) Ang2 to Ang1 ratio, *E*) high sensitivity C-reactive protein (Hs CRP), and *F*) fibrinogen from baseline to the end of the enrolment period on day 15 in participants on sildenafil and placebo. Between-group comparisons were adjusted for body mass index. Data are shown as median (Q1–Q3); *P < .05, **P < .01; ns = non-significant

Between groups, on day 15, ET-1 changed by −30% (−53 to 4) in the sildenafil group vs. −3% (−18 to 22) in the placebo group (*P* = .044, *Figure 3A*). Ang-2 changed by −5% (−11 to −4) with sildenafil compared to 3% (−5 to 15) with placebo (*P* = .035, *Figure 3B*), while ang-1 increased by 24% (17–54) with sildenafil vs. −4% (−19 to −2) with placebo (*P* = .001, *Figure 3C*), leading to a change in the ang-2/ang-1 ratio by −23% (−45 to −15), with sildenafil in comparison to 8% (−3 to 24) with placebo (*P* = .001, *Figure 3D*). No differences were noted in changes for hs-CRP and fibrinogen between participants receiving sildenafil and placebo (*Figure 3E and F*). After adjustment for body mass index, there remained a significant difference in percent change of ET-1 (*P* = .041), ang-2 (*P* = .039), ang-1 (*P* = .001), and ang-2/ang-1 ratio (*P* = .001) between participants on sildenafil and placebo.

**Figure 4:**
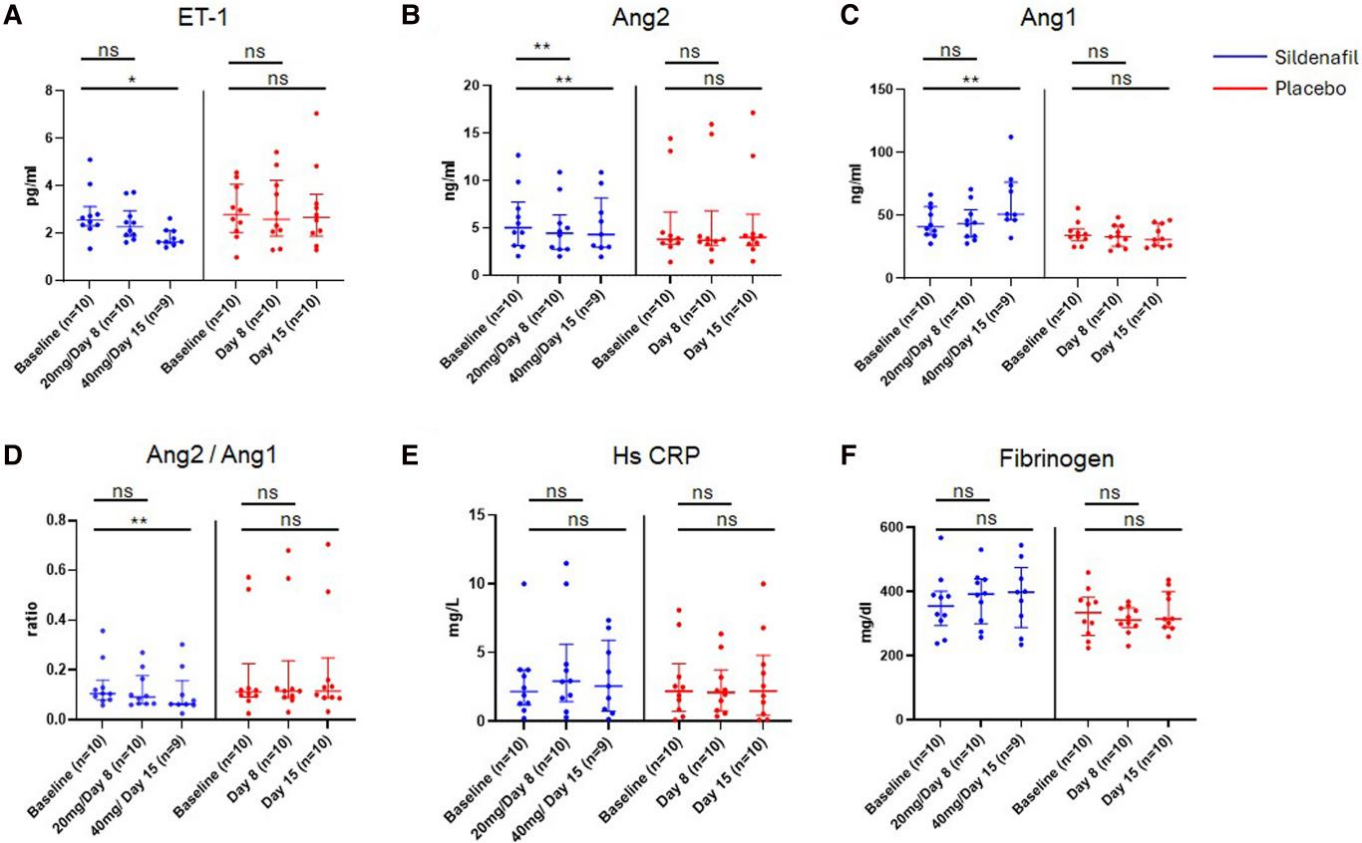
Within group changes in circulating levels of *A*) endothelin-1 (ET-1), *B*) angiopoietin (Ang) 2, *C*) angiopoietin (Ang) 1, *D*) Ang2 to Ang1 ratio, *E*) high-sensitivity C-reactive protein (Hs CRP), and *F*) fibrinogen for participants on sildenafil or placebo across study timepoints. Data are shown as median (Q1–Q3); *P < .05, **P < .01; ns = non-significant

Within groups, ET-1 was 2.6 (2.3–3.2 pg/ml) at baseline, 2.3 (1.9–3.0 pg/ml, *P* = .06 vs. baseline) with 20 mg sildenafil, and reduced to 1.6 (1.6–2.1 pg/ml, *P* = .039 vs. baseline) with 40 mg sildenafil (*Figure 4A*). Ang-2 was reduced from 5.0 (3.1–7.8) ng/ml to 4.4 (2.8–6.4 ng/ml, *P* = .002 vs. baseline) with 20 mg sildenafil and to 4.3 (3.0–8.2 ng/ml, *P* = .004 vs. baseline) with 40 mg sildenafil (*Figure 4B*). In contrast, ang-1 stayed similar from 41 (35–57 ng/ml) to 44 (33–55 ng/ml, *P* = 1.0 vs. baseline) with 20 mg sildenafil but rose to 51 (47–76 ng/ml, *P* = .004 vs. baseline) with 40 mg sildenafil (*Figure 4C*). This led to a reduction in the ang-2/ang-1 ratio from 0.10 (0.08–0.16) to 0.06 (0.06–0.16, *P* = .008 vs. baseline) with 40 mg sildenafil (*Figure 4D*). We noted no significant changes in hs-CRP from baseline (2.2, 1.2–3.7 mg/L) with either 20 mg (2.9, 1.4–5.6 mg/L, *P* = .44 vs. baseline) or 40 mg (2.6, 0.74–5.9 mg/L, *P* = .91 vs. baseline) sildenafil (*Figure 4E*). Fibrinogen also remained similar (baseline: 355, 294–401 mg/dl) to 392 (300–439 mg/dl *P* = .28 vs. baseline) with 20 mg and 398 (388–475 mg/dl, *P* = .50 vs. baseline) with 40 mg sildenafil (*Figure 4F*). Participants on placebo did not show changes in circulating markers.

### HeartMate 3 and sildenafil

The clinical characteristics of participants on HM 3 support assigned to sildenafil (*n* = 9) are noted in Supplementary Table S2. They were 55 (45–60) years old, 2 (22%) were female, and had been on HM 3 support for 349 (201–574) days prior to enrolment. Among these participants, collagen-induced platelet activation and aggregation changed from 86 (67–120 AUC) at baseline to 77 (54–117 AUC, *P* = .16 vs. baseline) with 20 mg sildenafil and were reduced to 59 (37–83 AUC, *P* = .015 vs. baseline) with 40 mg sildenafil (Supplementary Figure S4). TxA_2_ induced platelet activation, and aggregation was 83 (61–103 AUC) at baseline, 64 (41–119 AUC, *P* = .65 vs. baseline) with 20 mg sildenafil, and lowered to 63 (26–89 AUC, *P* = .039, Supplementary Figure S4) with 40 mg sildenafil. There were no significant changes in platelet activation and aggregation in patients receiving sildenafil with ADP in comparison to baseline (Supplementary Figure S4).

Amongst circulating mediators of vascular remodelling, ET-1 was 2.4 (2.3–3.4 pg/ml) at baseline, 2.1 (1.8–3.2 pg/ml, *P* = .13 vs. baseline) with 20 mg sildenafil, and was reduced to 1.6 (1.6–2.1 pg/ml, *P* = .02 vs. baseline) with 40 mg sildenafil (Supplementary Figure S5). Ang-2 was reduced from 5.5 (3.9- 8.4) ng/ml to 4.7 (2.9–7.3 ng/ml, *P* = .004 vs. baseline) with 20 mg sildenafil and further to 4.3 (3.0–8.2 ng/ml, *P* = .04 vs. baseline) with 40 mg sildenafil (Supplementary Figure S5). In contrast, ang-1 stayed similar from 42 (36–58 ng/ml) to 44 (32–58 ng/ml, *P* = 1.0 vs. baseline) with 20 mg sildenafil but increased to 51 (47–73 ng/ml, *P* = .004 vs. baseline) with 40 mg sildenafil (Supplementary Figure S5). This led to a reduction in the ang-2/ang-1 ratio from 0.11 (0.09–0.19) to 0.07 (0.06–0.19, *P* = .02 vs. baseline) with 40 mg sildenafil (Supplementary Figure S5). No significant changes were noted in hs-CRP and fibrinogen levels in comparison to baseline.

## Discussion

The principal findings of this investigation in outpatients on durable LVAD support are as follows: (i) 40 mg of sildenafil is well-tolerated by participants, (ii) platelet activation by collagen is reduced with sildenafil, (iii) mediators of vascular remodelling including ET-1, ang-2, and ang-2 to ang-1 ratio are lowered by sildenafil, and (iv) inflammatory biomarkers including hs-CRP and fibrinogen do not change with sildenafil in comparison to placebo. Within the subgroup of participants on HM 3 support assigned to sildenafil, platelet activation by collagen and TxA_2_, as well as circulating levels of ang-2 and ang-2 to ang-1 ratio, were reduced in comparison to baseline. These findings show that 15-day sildenafil exposure during LVAD support modifies platelet activation and reduces mediators of adverse vascular remodelling.

Activated platelets lead to clot formation, propagation of vascular disease, and ischaemic stroke.^20,21^ Studies have revealed that platelets are hyperactivated in patients on LVAD support with upregulation of P-selectin and adhesion pathways.^22–24^ Evidence suggests that this heightened level of platelet activation is due to device-induced shear stress,^25^ low levels of haemolysis,^14,26^ platelet interaction with activated monocytes, and inflamed endothelium.^24,27^ Sildenafil increases NO signalling in platelets by reducing the hydrolysis of cyclic guanosine monophosphate (cGMP) by inhibiting PDE5, which leads to platelet inhibition.^10^ Indeed, our findings show a modest reduction in platelet activation upon agonism by collagen with sildenafil administration during LVAD support. These mild inhibitory effects on platelets with sildenafil have been shown in other populations. In patients with coronary artery disease, Halcox *et al.* showed a ∼20% reduction in the expression of activated IIB/IIIA receptor on platelets after ADP stimulation with administration of sildenafil.^7^ Akand and colleagues also noted a modest reduction in platelet activation with sildenafil in patients with erectile dysfunction.^28^ While other mechanistic studies have noted that sildenafil reduces platelet aggregation and intimal hyperplasia following vascular injury through the cGMP-dependent protein kinase pathway.^8^ It is notable that aspirin, which exerts strong anti-platelet effects, as well as irritation of the gastrointestinal mucosa, led to more bleeding events during magnetically levitated LVAD support, and its avoidance was not inferior in preventing hemocompatibility-related adverse events, including thrombosis and ischaemic stroke.^29^ While it remains unknown if the observed modification of platelet activation by sildenafil during LVAD support, which needs to be cautiously interpreted in the setting of study truncation, will reduce thrombogenic events without increasing bleeding, its favourable effects appear to extend into the vasculature.

There is rapid onset vascular remodelling in the aorta after placement of continuous flow LVADs. Ambardekar *et al.* noted an over 2-fold increase in adventitial thickness and collagen deposition within the aortic wall, which were apparent as early as 2 months after LVAD implantation.^5^ Similar changes of increased vascular stiffness and reduced distensibility are also observed in cerebrovascular vessels, including the carotid arteries.^30,31^ Although the mechanisms by which low pulsatile conditions induced by LVADs cause macrovascular remodelling are uncertain, a key mediator of fibrosis, ET-1, could be at least partially implicated. ET-1 is a circulating ligand peptide of ET_A_ and ET_B_ receptors. ET-1 is produced by endothelial cells as a potent vasoconstrictor to regulate vasomotor function but also triggers vascular fibrosis by increasing the synthesis and deposition of components of the extracellular matrix (ECM) through activation of myofibroblasts derived from pericytes.^32^ Prior studies have noted an increase in ET-1 after LVAD placement, with an acute reduction by administration of inhaled NO.^33^ Our findings also show a reduction in ET-1 levels by sildenafil, which enhances NO signalling. Similar reductions in ET-1 with sildenafil have been noted in patients with erectile dysfunction^34^ and small, randomized studies show improvements in vascular stiffness in non-LVAD populations.^35–37^ Although the Phosphodiesterase-5 Inhibition to Improve Clinical Status and Exercise Capacity in Heart Failure with Preserved Ejection Fraction (RELAX) trial failed to show improved exercise capacity at 6 months with sildenafil for patients with heart failure and preserved ejection fraction,^38^ its mechanistic sub-study showed beneficial vascular effects with reduced arterial elastance and endothelial dysfunction.^39^ Our findings of ET-1 reduction as a secondary measure are only hypothesis-generating, and it remains uncertain if longer-term administration of sildenafil during LVAD support can indeed lead to a reduction in macrovascular remodelling in aortic and cerebrovascular beds.

Nonsurgical bleeding during LVAD support can manifest as haemorrhagic stroke or, more commonly, mucosal gastrointestinal bleeding. Microvascular angioectasias in the gastrointestinal tract are commonly noted on video capsule endoscopy, as well as in the nasopharynx, are typical sites of nonsurgical bleeding during LVAD support.^40^ Moreover, subclinical cerebral microhemorrhages with associated vascular inflammation have also been detected after LVAD explantation.^41,42^ Evidence suggests that angioectasia formation and propagation after LVAD placement occur due to dysregulated angiogenesis mediated by altered levels of ang-1 and ang-2.^6^ These proteins induce angiogenesis by acting as competitors of Tie-2, a receptor tyrosine kinase on the surface of endothelial cells. Ang-1 is induced by perivascular cells and, together with vascular endothelial growth factor (VEGF), promotes vessel development and stability.^43^ In contrast, ang-2, which is stored in Weibel-Palade bodies within endothelial cells, antagonizes Tie-2/ang1 to yield abnormal vascular growth associated with inflammation.^44^ Following LVAD placement, increased endothelial expression and higher serum levels of ang-2 increase the ang-2/ang-1 ratio, leading to the formation of dysplastic angioectasias.^6^  *Ex vivo* studies show a positive linear correlation between serum concentration of ang-2 and tube cell formation with abrogation of this effect by ang-2 antibody blockade.^6^ Moreover, pharmacological agents that inhibit ang-2 pathways, including digoxin^45^ and angiotensin-converting enzyme inhibitors,^46^ are associated with a lower incidence of gastrointestinal bleeding in LVAD patients. Previous randomized studies in patients with diabetes mellitus have noted that sildenafil leads to a reduction in the ang-2/ang-1 ratio.^47^ Our findings show similar effects with sildenafil administration during LVAD support as a near 40% within-group reduction in the ang-2/ang-1 ratio. These findings remain hypothesis-generating, and the mechanism by which sildenafil lowers ang-2/ang-1 merits further investigation, as this pharmacological approach may provide a method for limiting aberrant angiodysplasia formation to potentially reduce microvascular remodelling and bleeding outcomes.

Collectively, our findings of lower platelet activation and mediators of vascular remodelling pathways with sildenafil administration provide credence to large-scale observational studies from INTERMACS, which showed a lower incidence of ischaemic stroke and mortality during sildenafil use across various LVADs.^16^ It is notable that a separate INTERMACS analysis showed neutral associations of PDE5i with these outcomes,^48^ but this analysis was limited by not including contemporary devices and era. Moreover, INTERMACS analyses noting an association between sildenafil use and gastrointestinal bleeding are limited by an indication bias in assessing this particular outcome.^48^ This confounding by indication arises as those receiving sildenafil are typically prescribed this therapy due to right heart ventricular dysfunction, which in itself is a risk factor for gastrointestinal bleeding.^49,50^

## Limitations

The impact of study truncation on endpoint measures is uncertain and may inflate the effect size. The findings are at least partially informed by a 95% conditional power for reduced collagen-induced platelet activation during sildenafil administration if the study reached full enrolment, but must be cautiously interpreted in the setting of reduced sample size. The sustainability of changes in platelet activation, ET-1, ang-1, and ang-2 with sildenafil is uncertain beyond the 15-day enrolment period. The study enrolled late and stable survivors of LVAD therapy, and it remains unknown whether similar effects on safety, tolerability, and study endpoints would be present in the early period after LVAD placement. This study was not designed to detect device-specific changes in platelet function and circulating mediators of vascular remodelling, and device-specific effects cannot be reliably assessed. Moreover, limited sample size precluded reliably powered post-hoc assessments of between-group changes in study measures in the HM 3 subgroup. Nonetheless, exploratory within-group changes do indicate that participants on HM 3 support had a reduction in platelet activation and mediators of vascular remodelling in comparison to baseline. Finally, clinical outcomes were not assessed in this study.

## Conclusion

In participants on durable LVAD support, short-term administration of sildenafil led to modification of platelet activation by collagen and reduction in mediators of vascular remodelling including ET-1, ang-2, and ang-2/ang-1 ratio. These findings must be interpreted with caution due to study truncation but provide hypothesis generation for further studies. Longer-term, randomized, mechanistic, and clinical studies will be needed to determine how sildenafil can impede large and small vessel remodelling during LVAD support.

## Supplementary Material

xvag099_Supplementary_Data

## Declarations

### Data Availability

No data were generated or analysed for this manuscript.

### Pre-registered Clinical Trial Number

None supplied.

## References

[1] Mehra MR, Uriel N, Naka Y, Cleveland JC Jr, Yuzefpolskaya M, Salerno CT, et al A fully magnetically levitated left ventricular assist device. N Engl J Med 2019;380:1618–27. 10.1056/NEJMoa1900486 30883052

[2] Jorde UP, Saeed O, Koehl D, Morris AA, Wood KL, Meyer DM, et al The Society of Thoracic Surgeons Intermacs 2023 annual report: focus on magnetically levitated devices. Ann Thorac Surg 2024;117:33–44. 10.1016/j.athoracsur.2023.11.004 37944655

[3] Colombo PC, Mehra MR, Goldstein DJ, Estep JD, Salerno C, Jorde UP, et al Comprehensive analysis of stroke in the long-term cohort of the MOMENTUM 3 study: a randomized controlled trial of the HeartMate 3 versus the HeartMate II cardiac pump. Circulation 2019;139:155–68. 10.1161/CIRCULATIONAHA.118.037231 30586698

[4] Vidula H, Takeda K, Estep JD, Silvestry SC, Milano C, Cleveland JC Jr, et al Hospitalization patterns and impact of a magnetically-levitated left ventricular assist device in the MOMENTUM 3 trial. J Heart Failure 2022;10:470–81. 10.1016/j.jchf.2022.03.007 35772857

[5] Ambardekar AV, Stratton MS, Dobrinskikh E, Hunter KS, Tatman PD, Lemieux ME, et al Matrix-degrading enzyme expression and aortic fibrosis during continuous-flow left ventricular mechanical support. J Am Coll Cardiol 2021;78:1782–95. 10.1016/j.jacc.2021.08.047 34711337 PMC8562886

[6] Tabit CE, Chen P, Kim GH, Fedson SE, Sayer G, Coplan MJ, et al Elevated angiopoietin-2 level in patients with continuous-flow left ventricular assist devices leads to altered angiogenesis and is associated with higher nonsurgical bleeding. Circulation 2016;134:141–52. 10.1161/CIRCULATIONAHA.115.019692 27354285 PMC4942355

[7] Halcox JP, Nour KR, Zalos G, Mincemoyer R, Waclawiw MA, Rivera CE, et al The effect of sildenafil on human vascular function, platelet activation, and myocardial ischemia. J Am Coll Cardiol 2002;40:1232–40. 10.1016/S0735-1097(02)02139-3 12383570

[8] Yang H-M, Jin S, Jang H, Kim J-Y, Lee J-E, Kim J, et al Sildenafil reduces neointimal hyperplasia after angioplasty and inhibits platelet aggregation via activation of cGMP-dependent protein kinase. Sci Rep 2019;9:7769. 10.1038/s41598-019-44190-7 31123275 PMC6533301

[9] Villagra J, Shiva S, Hunter LA, Machado RF, Gladwin MT, Kato GJ. Platelet activation in patients with sickle disease, hemolysis-associated pulmonary hypertension, and nitric oxide scavenging by cell-free hemoglobin. Blood 2007;110:2166–72. 10.1182/blood-2006-12-061697 17536019 PMC1976348

[10] Sandner P, Hütter J, Tinel H, Ziegelbauer K, Bischoff E. PDE5 inhibitors beyond erectile dysfunction. Int J Impot Res 2007;19:533–43. 10.1038/sj.ijir.3901577 17625575

[11] Ferrini M, Kovanecz I, Sanchez S, Vernet D, Davila H, Rajfer J, et al Long-term continuous treatment with sildenafil ameliorates aging-related erectile dysfunction and the underlying corporal fibrosis in the rat. Biol Reprod 2007;76:915–23. 10.1095/biolreprod.106.059642 17287493

[12] Wen JJ, Cummins C, Radhakrishnan RS. Sildenafil recovers burn-induced cardiomyopathy. Cells 2020;9:1393. 10.3390/cells9061393 32503314 PMC7349507

[13] Milara J, Escrivá J, Ortiz JL, Juan G, Artigues E, Morcillo E, et al Vascular effects of sildenafil in patients with pulmonary fibrosis and pulmonary hypertension: an ex vivo/in vitro study. Eur Respir J 2016;47:1737–49. 10.1183/13993003.01259-2015 27009174

[14] Saeed O, Rangasamy S, Selevany I, Madan S, Fertel J, Eisenberg R, et al Sildenafil is associated with reduced device thrombosis and ischemic stroke despite low-level hemolysis on Heart Mate II support. Circ Heart Fail 2017;10:e004222. 10.1161/CIRCHEARTFAILURE.117.004222 29092891

[15] Xanthopoulos A, Tryposkiadis K, Triposkiadis F, Fukamachi K, Soltesz EG, Young JB, et al Postimplant phosphodiesterase type 5 inhibitors use is associated with lower rates of thrombotic events after left ventricular assist device implantation. J Am Heart Assoc 2020;9:e015897. 10.1161/JAHA.119.015897 32648508 PMC7660717

[16] Xanthopoulos A, Wolski K, Wang Q, Blackstone EH, Randhawa VK, Soltesz EG, et al Postimplant phosphodiesterase-5 inhibitor use in centrifugal flow left ventricular assist devices. JACC Heart Fail 2022;10:89–100. 10.1016/j.jchf.2021.09.008 35115092

[17] McGlasson DL, Fritsma GA. Whole blood platelet aggregometry and platelet function testing. Semin Thromb Hemost 2009;35:168–80. 10.1055/s-0029-1220325 19408190

[18] Flanders MM, Crist R, Rodgers GM. Comparison of five thrombin time reagents. Clin Chem 2003;49:169–72. 10.1373/49.1.169 12507975

[19] Hyndman RJ, Fan Y. Sample quantiles in statistical packages. Am Stat 1996;50:361–5. 10.1080/00031305.1996.10473566

[20] Hackam DG, Spence JD. Antiplatelet therapy in ischemic stroke and transient ischemic attack: an overview of major trials and meta-analyses. Stroke 2019;50:773–8. 10.1161/STROKEAHA.118.023954 30626286

[21] Lebas H, Yahiaoui K, Martos R, Boulaftali Y. Platelets are at the nexus of vascular diseases. Front Cardiovasc Med 2019;6:132. 10.3389/fcvm.2019.00132 31572732 PMC6749018

[22] Radovancevic R, Matijevic N, Bracey AW, Radovancevic B, Elayda M, Gregoric ID, et al Increased leukocyte-platelet interactions during circulatory support with left ventricular assist devices. ASAIO J 2009;55:459–64. 10.1097/MAT.0b013e3181b235af 19730003

[23] Griesshammer M, Beneke H, Nussbaumer B, Grünewald M, Bangerter M, Bergmann L. Increased platelet surface expression of P-selectin and thrombospondin as markers of platelet activation in essential thrombocythaemia. Thromb Res 1999;96:191–6. 10.1016/S0049-3848(99)00095-X 10588461

[24] Frey C, Koliopoulou AG, Montenont E, Tolley ND, Javan H, McKellar SH, et al Longitudinal assessment of the platelet transcriptome in advanced heart failure patients following mechanical unloading. Platelets 2020;31:952–9. 10.1080/09537104.2020.1714573 31934818 PMC7358121

[25] De Biasi AR, Manning KB, Salemi A. Science for surgeons: understanding pump thrombogenesis in continuous-flow left ventricular assist devices. J Thorac Cardiovasc Surg 2015;149:667–73. 10.1016/j.jtcvs.2014.11.041 25534307

[26] Tran PL, Pietropaolo M-G, Valerio L, Brengle W, Wong RK, Kazui T, et al Hemolysate-mediated platelet aggregation: an additional risk mechanism contributing to thrombosis of continuous flow ventricular assist devices. Perfusion 2016;31:401–8. 10.1177/0267659115615206 26590166 PMC4874907

[27] Granja T, Magunia H, Schüssel P, Fischer C, Pruefer T, Schibilsky D, et al Left ventricular assist device implantation causes platelet dysfunction and proinflammatory platelet-neutrophil interaction. Platelets 2022;33:132–40. 10.1080/09537104.2020.1859101 33347335

[28] Akand M, Gencer E, Yaman Ö, Erişgen G, Tekin D, Özdiler E. Effect of sildenafil on platelet function and platelet cGMP of patients with erectile dysfunction. Andrologia 2015;47:1098–102. 10.1111/and.12387 25486996

[29] Mehra MR, Netuka I, Uriel N, Katz JN, Pagani FD, Jorde UP, et al Aspirin and hemocompatibility events with a left ventricular assist device in advanced heart failure: the ARIES-HM3 randomized clinical trial. JAMA 2023;330:2171–81. 10.1001/jama.2023.23204 37950897 PMC10640705

[30] Templeton DL, John R, Painter P, Kelly AS, Dengel DR. Effects of the left ventricular assist device on the compliance and distensibility of the carotid artery. Heart vessels 2013;28:377–84. 10.1007/s00380-012-0271-4 22875409

[31] Kiyatkin M, Stöhr E, Zuver A, Gaudig A, Yuzefpolskaya M, Colombo P, et al Carotid artery flow and its assciation with stroke during left ventricular assist device support. Crit Care Med 2019;47:53. 10.1097/01.ccm.0000550897.64734.66

[32] Rodríguez-Pascual F, Busnadiego O, González-Santamaría J. The profibrotic role of endothelin-1: is the door still open for the treatment of fibrotic diseases? J Life Sciences 2014;118:156–64. 10.1016/j.lfs.2013.12.024 24378671

[33] Wagner FD, Buz S, Knosalla C, Hetzer R, Hocher B. Modulation of circulating endothelin-1 and big endothelin by nitric oxide inhalation following left ventricular assist device implantation. Circulation 2003;108:II278–84. 10.1161/01.cir.0000090630.48893.70 12970246

[34] Angelis K, Konstantinos G, Anastasios A, Dionisios S, Petros P. The impact of daily sildenafil on levels of soluble molecular markers of endothelial function in plasma in patients with erectile dysfunction. Expert Opin Pharmacother 2009;10:155–60. 10.1517/14656560802678211 19236190

[35] Vlachopoulos C, Hirata K, O’Rourke MF. Effect of sildenafil on arterial stiffness and wave reflection. Vascular Medicine 2003;8:243–8. 10.1191/1358863x03vm509ra 15125484

[36] Vlachopoulos C, Terentes-Printzios D, Ioakeimidis N, Rokkas K, Samentzas A, Aggelis A, et al Beneficial effect of vardenafil on aortic stiffness and wave reflections. J Clin Pharmacol 2012;52:1215–21. 10.1177/0091270011413586 21953573

[37] Aversa A, Letizia C, Francomano D, Bruzziches R, Natali M, Lenzi A. A spontaneous, double-blind, double-dummy cross-over study on the effects of daily vardenafil on arterial stiffness in patients with vasculogenic erectile dysfunction. Int J Cardiol 2012;160:187–91. 10.1016/j.ijcard.2011.04.003 21546099

[38] Redfield MM, Chen HH, Borlaug BA, Semigran MJ, Lee KL, Lewis G, et al Effect of phosphodiesterase-5 inhibition on exercise capacity and clinical status in heart failure with preserved ejection fraction: a randomized clinical trial. JAMA 2013;309:1268–77. 10.1001/jama.2013.2024 23478662 PMC3835156

[39] Borlaug BA, Lewis GD, McNulty SE, Semigran MJ, LeWinter M, Chen H, et al Effects of sildenafil on ventricular and vascular function in heart failure with preserved ejection fraction. Circ Heart Fail 2015;8:533–41. 10.1161/CIRCHEARTFAILURE.114.001915 25782985 PMC4439337

[40] Patel SR, Madan S, Saeed O, Algodi M, Luke A, Gibber M, et al Association of nasal mucosal vascular alterations, gastrointestinal arteriovenous malformations, and bleeding in patients with continuous-flow left ventricular assist devices. JACC Heart Fail 2016;4:962–70. 10.1016/j.jchf.2016.08.005 27744088

[41] Murase S, Okazaki S, Yoshioka D, Watanabe K, Gon Y, Todo K, et al Abnormalities of brain imaging in patients after left ventricular assist device support following explantation. J Heart Lung Transplant. 2020;39:220–7. 10.1016/j.healun.2019.11.019 31843457

[42] Fan TH, Cho S-M, Prayson RA, Hassett CE, Starling RC, Uchino K. Cerebral microvascular injury in patients with left ventricular assist device: a neuropathological study. Transl Stroke Res 2022;13:257–64. 10.1007/s12975-021-00935-z 34494179 PMC9439715

[43] Cascone T, Heymach JV. Targeting the angiopoietin/Tie2 pathway: cutting tumor vessels with a double-edged sword? J Clin Oncol 2011;30:441–4. 10.1200/JCO.2011.38.7621 22184396

[44] Metcalf DJ, Nightingale TD, Zenner HL, Lui-Roberts WW, Cutler DF. Formation and function of Weibel-Palade bodies. J Cell Sci 2008;121:19–27. 10.1242/jcs.03494 18096688

[45] Vukelic S, Vlismas PP, Patel SR, Xue X, Shitole SG, Saeed O, et al Digoxin is associated with a decreased incidence of angiodysplasia-related gastrointestinal bleeding in patients with continuous-flow left ventricular assist devices. Circ Heart Fail 2018;11:e004899. 10.1161/CIRCHEARTFAILURE.118.004899 30354557

[46] Converse MP, Sobhanian M, Taber DJ, Houston BA, Meadows HB, Uber WE. Effect of angiotensin II inhibitors on gastrointestinal bleeding in patients with left ventricular assist devices. J Am Coll Cardiol 2019;73:1769–78. 10.1016/j.jacc.2019.01.051 30975293

[47] Venneri MA, Barbagallo F, Fiore D, De Gaetano R, Giannetta E, Sbardella E, et al PDE5 inhibition stimulates Tie2-expressing monocytes and angiopoietin-1 restoring angiogenic homeostasis in diabetes. J Clin Endocrinol Metab 2019;104:2623–36. 10.1210/jc.2018-02525 31102457

[48] Grandin EW, Gulati G, Nunez JI, Kennedy K, Rame JE, Atluri P, et al Outcomes with phosphodiesterase-5 inhibitor use after left ventricular assist device: an STS-INTERMACS analysis. Circ Heart Fail 2022;15:e008613. 10.1161/CIRCHEARTFAILURE.121.008613 35332780 PMC9205418

[49] Liebo M, Newman J, Yu M, Hussain Z, Malik S, Lowes B, et al Preoperative right heart dysfunction and gastrointestinal bleeding in patients with left ventricular assist devices. ASAIO Journal 2021;67:324–31. 10.1097/MAT.0000000000001224 33627608

[50] Sparrow CT, Nassif ME, Raymer DS, Novak E, LaRue SJ, Schilling JD. Pre-operative right ventricular dysfunction is associated with gastrointestinal bleeding in patients supported with continuous-flow left ventricular assist devices. JACC Heart Fail 2015;3:956–64. 10.1016/j.jchf.2015.09.009 26577618

