# Supplementary material for: Effect of sildenafil on platelet activation and mediators of vascular remodelling during LVAD support
Source: ESC Heart Fail. 2026 Apr 2;13(3):xvag099. doi: 10.1093/eschf/xvag099 (PMC13175256; doi:10.1093/eschf/xvag099)
Supplement: xvag099_Supplementary_Data [file xvag099_supplementary_data.docx]

**Supplemental Tables and Figures**

**Supplemental Figure 1:** A) Between group changes in INR and B) warfarin dose from baseline to the end of the enrollment period on day 15 in participants on sildenafil and placebo. ns=non-significant.

1. **B)**

**
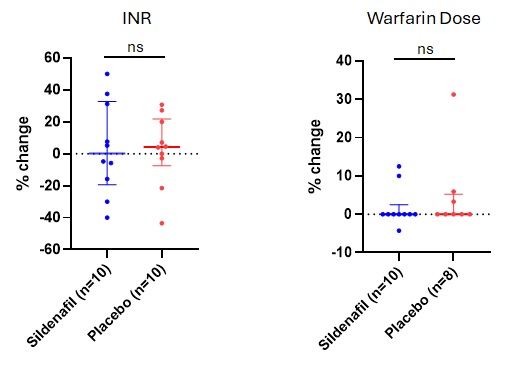
**

**Supplemental Figure 2**: A) Between group changes in doppler blood pressure (DopBP) from baseline to the end of the enrollment period on day 15 in participants on sildenafil and placebo.


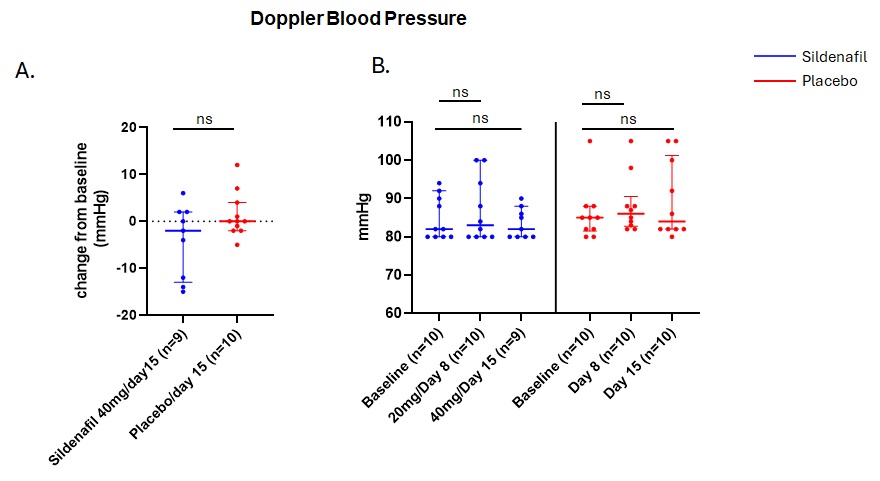


**Supplemental Figure 3:** Changes in platelet activation and aggregation after addition of arachidonic acid in participants on and off aspirin.


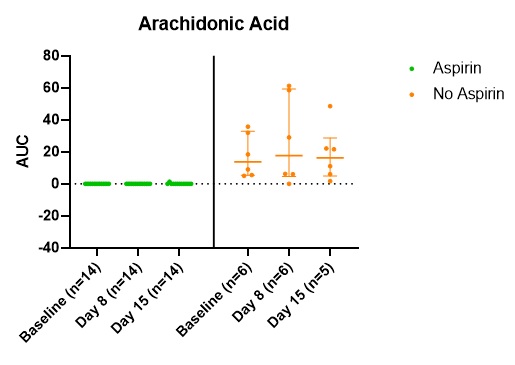


**Supplemental Figure 4:** Within group changes in platelet activation and aggregation across study timepoints in participants on HeartMate 3 support assigned to sildenafil. Platelet activation and aggregation was measured by ex-vivo whole blood aggregometry with agonism from **A**) collagen, **B**) thromboxane A_2_, and **C**) adenosine diphosphate and is shown as area under the curve (AUC). Data are shown as median (Q1-Q3), *=p<0.05, ns=non-significant in comparison to baseline values.


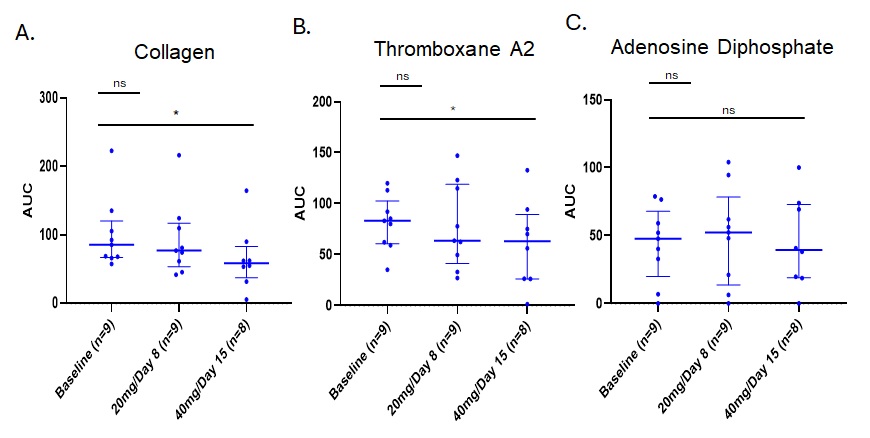


**Supplemental Figure 5:** Within group changes in circulating levels of **A**) endothelin-1 (ET-1), **B**) angiopoietin (Ang) 2, **C**) angiopoietin (Ang) 1, **D**) Ang2 to Ang1 ratio, **E**) high sensitivity C-reactive protein (Hs CRP), and **F**) fibrinogen for participants on HeartMate 3 support assigned to sildenafil across study timepoints. Data are shown as median (Q1-Q3); *=p<0.05, **=p<0.01; ns=non-significant in comparison to baseline values.


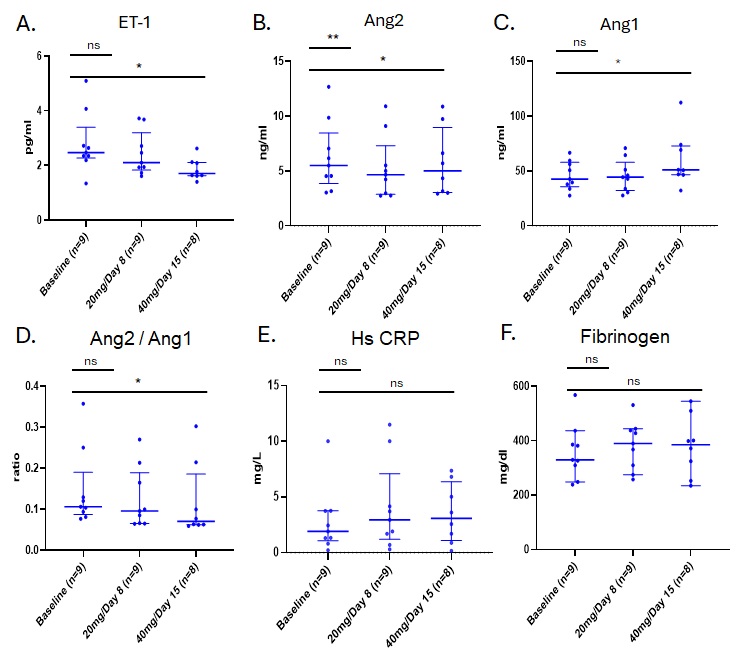


**Supplemental Table 1**: Background heart failure medical therapy and anti-hypertensive medical therapy during the 15-day enrollment period.

| No. | Group | Baseline | Day 8 | Day 15 |
| --- | --- | --- | --- | --- |
| 1 | Sildenafil | Carvedilol 25mg every 12 hours  Lisinopril 10mg once daily | Carvedilol 25mg every 12 hours  Lisinopril 10mg once daily | Carvedilol 25mg every 12 hours  Lisinopril 10mg once daily |
| 2 | Sildenafil | Toprol XL 200mg once daily  Enalapril 10mg every 12 hours  Nifedipine 60mg once daily | Toprol XL 200mg once daily  Enalapril 10mg every 12 hours  Nifedipine 60mg once daily | Toprol XL 200mg once daily  Enalapril 10mg every 12 hours  Nifedipine 60mg once daily |
| 3 | Sildenafil | Lisinopril 5mg once daily  Coreg 6.25mg every 12 hours | Lisinopril 5mg once daily  Coreg 6.25mg every 12 hours | Lisinopril 5mg once daily  Coreg 6.25mg every 12 hours |
| 4 | Sildenafil | Toprol XL 100 mg daily  Nifedipine 30mg once daily | Toprol XL 100 mg daily  Nifedipine 30mg once daily | Toprol XL 100 mg daily  Nifedipine 30mg once daily |
| 5 | Sildenafil | Carvedilol 3.125mg every 12 hours  Ramipril 2.5mg once daily | Carvedilol 3.125mg every 12 hours  Ramipril 2.5mg once daily | Carvedilol 3.125mg every 12 hours  Ramipril 2.5mg once daily |
| 6 | Sildenafil | None | None | None |
| 7 | Sildenafil | Lisinopril 5mg once daily  Spironolactone 25mg once daily | Lisinopril 5mg once daily  Spironolactone 25mg once daily | Lisinopril 5mg once daily  Spironolactone 25mg once daily |
| 8 | Sildenafil | Carvedilol 3.125mg every 12 hours  Spironolactone 25mg once daily  Hydralazine 100mg every 8 hours | Carvedilol 3.125mg every 12 hours  Spironolactone 25mg once daily  Hydralazine 100mg every 8 hours | Carvedilol 3.125mg every 12 hours  Spironolactone 25mg once daily  Hydralazine 100mg every 8 hours |
| 9 | Sildenafil | Toprol XL 37.5mg once daily  Losartan 25mg once daily | Toprol XL 37.5mg once daily  Losartan 25mg once daily | Toprol XL 37.5mg once daily  Losartan 25mg once daily |
| 10 | Sildenafil | Carvedilol 25mg every 12 hours  Losartan 100mg once daily  Hydralazine 100mg every 8 hours | Carvedilol 25mg every 12 hours  Losartan 100mg once daily  Hydralazine 100mg every 8 hours | Carvedilol 25mg every 12 hours  Losartan 100mg once daily  Hydralazine 100mg every 8 hours |
| 11 | Placebo | Toprol XL 200mg every day  Amlodipine 10mg once daily | Toprol XL 200mg every day  Amlodipine 10mg once daily | Toprol XL 200mg every day  Amlodipine 10mg once daily |
| 12 | Placebo | Carvedilol 3.125mg every 12 hours | Carvedilol 3.125mg every 12 hours | Carvedilol 3.125mg every 12 hours |
| 13 | Placebo | Carvedilol 12.5mg every 12 hours  Lisinopril 5mg once daily | Carvedilol 12.5mg every 12 hours  Lisinopril 5mg once daily | Carvedilol 12.5mg every 12 hours  Lisinopril 5mg once daily |
| 14 | Placebo | Toprol XL 25 mg once daily  Losartan 25mg once daily | Toprol XL 25 mg once daily  Losartan 25mg once daily | Toprol XL 25 mg once daily  Losartan 25mg once daily |
| 15 | Placebo | Carvedilol 3.125 every 12 hours | Carvedilol 3.125 every 12 hours | Carvedilol 3.125 every 12 hours |
| 16 | Placebo | Carvedilol 12.5mg every 12 hours  Hydralazine 50mg every 8 hours  Amlodipine 10mg once daily | Carvedilol 12.5mg every 12 hours  Hydralazine 50mg every 8 hours  Amlodipine 10mg once daily | Carvedilol 12.5mg every 12 hours  Hydralazine 50mg every 8 hours  Amlodipine 10mg once daily |
| 17 | Placebo | Carvedilol 12.5mg every 12 hours | Carvedilol 12.5mg every 12 hours | Carvedilol 12.5mg every 12 hours |
| 18 | Placebo | Toprol XL 75mg once daily  Lisinopril 20mg once daily  Hydralazine 50mg every 8 hours | Toprol XL 75mg once daily  Lisinopril 20mg once daily  **Hydralazine 75mg every 8 hours** | Toprol XL 75mg once daily  Lisinopril 20mg once daily  Hydralazine 75mg every 8 hours |
| 19 | Placebo | Toprol XL 150mg once daily  Losartan 25mg daily  Spironolactone 25mg daily | Toprol XL 150mg once daily  Losartan 25mg daily  Spironolactone 25mg daily | Toprol XL 150mg once daily  Losartan 25mg daily  Spironolactone 25mg daily |
| 20 | Placebo | Toprol XL 100mg once daily  Spironolactone 25mg daily | Toprol XL 100mg once daily  Spironolactone 25mg daily | Toprol XL 100mg once daily  Spironolactone 25mg daily |

**Supplemental Table 2**: Baseline Characteristics of study participants on Heart Mate 3 support assigned to sildenafil.

| HM 3 Participants on sildenafil | (n=9) |
| --- | --- |
| Age (years) | 55 (45-60) |
| Female (n, %) | 2 (22) |
| Body Mass Index (Kg/m2) | 32 (25-41) |
| Ischemic Cardiomyopathy (n, %) | 2 (22) |
| Diabetes Mellitus (n, %) | 4 (44) |
| History of stroke (n, %) | 2 (22) |
| Race / ethnicity (n, %) | NHB: 4 (44)  NHW: 1 (11)  H: 4 (44) |
| LVAD duration (days) | 349 (201-574) |
| History of HRAE during LVAD (n, %) | 2 (22) |
| Aspirin (n, %) | 7 (78) |
| INR (IU) | 2.3 (1.9-2.5) |
| eGFR (ml/min/BSA) | 72 (53-89) |
| Lactate Dehydrogenase (U/l) | 240 (233-275) |
| Hemoglobin (g/dl) | 13.2 (12.5-13.6) |
| Platelets (k/ul) | 245 (169-293) |
| White blood cell (k/ul) | 8.3 (5.5-9.3) |
| Haptoglobin (mg/dl) | 114 (59-138) |

*p value for sildenafil vs. placebo; LVAD=left ventricular assist device; NHB=non-Hispanic black, NHW=non-Hispanic white, H=Hispanic; HRAE=Hemocompatibility Related Adverse Events; INR=international normalized ratio; GFR=glomerular filtration rate; BSA=body surface area
